# Risk and Secondary Prevention of Stroke Recurrence: A Population-Base Cohort Study

**DOI:** 10.1161/STROKEAHA.120.028992

**Published:** 2020-07-10

**Authors:** Clare Flach, Walter Muruet, Charles D.A. Wolfe, Ajay Bhalla, Abdel Douiri

**Affiliations:** 1 King’s College London, School of Population Health and Environmental Sciences, United Kingdom (C.F., W.M., C.D.A.W., A.B., A.D.).; 2 National Institute for Health Research Applied Research Collaboration (ARC) South London (C.D.A.W., A.D.), Guy’s and St Thomas’ National Health Service Foundation Trust and King’s College London, United Kingdom.; 3 Department of Ageing Health and Stroke (A.B.), Guy’s and St Thomas’ National Health Service Foundation Trust and King’s College London, United Kingdom.

**Keywords:** atrial fibrillation, prevalence, risk factors, secondary prevention

## Abstract

**Background and Purpose:**

With recent advances in secondary prevention management, stroke recurrence rates may have changed substantially. We aim to estimate risks and trends of stroke recurrence over the past 2 decades in a population-based cohort of patients with stroke.

**Methods:**

Patients with a first-ever stroke between 1995 and 2018 in South London, United Kingdom (n=6052) were collected and analyzed. Rates of recurrent stroke with 95% CIs were stratified by 5-year period of index stroke and etiologic TOAST (Trial of ORG 10172 in Acute Stroke Treatment) subtype. Cumulative incidences were estimated and multivariate Cox models applied to examine associations of recurrence and recurrence-free survival.

**Results:**

The rate of stroke recurrence at 5 years reduced from 18% (95% CI, 15%–21%) in those who had their stroke in 1995 to 1999 to 12% (10%–15%) in 2000 to 2005, and no improvement since. Recurrence-free survival has improved (35%, 1995–1999; 67%, 2010–2015). Risk of recurrence or death is lowest for small-vessel occlusion strokes and other ischemic causes (36% and 27% at 5 years, respectively). For cardioembolic and hemorrhagic index strokes around half of first recurrences are of the same type (54% and 51%, respectively). Over the whole study period a 54% increased risk of recurrence was observed among those who had atrial fibrillation before the index stroke (hazard ratio, 1.54 [1.09–2.17]).

**Conclusions:**

The rate of recurrence reduced until mid-2000s but has not changed over the last decade. The majority of cardioembolic or hemorrhagic strokes that have a recurrence are stroke of the same type indicating that the implementation of effective preventive strategies is still suboptimal in these stroke subtypes.

## Abstract 2

Supplemental Digital Content is available in the text.

It is estimated from a global meta-analysis in 2011 that 11% of individuals will have a recurrence within a year of their first stroke and 26% within 5 years. However, most of the studies included (12/13) provided follow-up to only 1-year poststroke with only 7 having information on recurrence to 5 years.The authors report that the rate of recurrence at 5 years halved over the period of the studies (1961–2006).^1^

Furthermore, systematic reviews report that secondary prevention measures (antithrombotic, statin, and antihypertensive therapies) can reduce the risk of secondary vascular events by 20% to 30%.^2–5^ This suggests that there may have been large improvements in recent years in reducing recurrence after stroke. Observational studies have also described this pattern through the 1990s and 2000s.^6–10^ However, with the increasing prevalence of risk factors such as diabetes mellitus as well as suboptimal uptake of preventative therapies^11,12^ these improvements may be at risk.

There is some evidence that the type of initial stroke has an impact on recurrence,^13,14^ though this is not shown in all studies,^15^ mainly because of limitation of the sample size. Cardioembolic strokes were observed to have the greatest risk for recurrence while individuals with lacunar stroke and hemorrhagic stroke have a smaller risk for recurrent stroke^13^ but higher risk of death compared with nonischemic strokes.^16^ There is a need to understand further the association between initial stroke and subsequent stroke to be able to recommend more tailored prevention and monitoring strategies to reduce the risk of stroke recurrence.

In this study, we examine risks and trends over the past 23 years, of recurrence and death after stroke in a population-based study and identify patient groups at risk of stroke recurrence including age, sex, and etiologic subtype.

## Methods

Because of the sensitive nature of the data collected for this study, requests to access the data set from qualified researchers trained in human subject confidentiality protocols may be requested through the below link: https://www.kcl.ac.uk/lsm/research/divisions/hscr/research/groups/stroke/index.aspx

### Study Population

The South London Stroke Register (SLSR), established in 1995, is an ongoing multi-ethnic, urban-based population register of all first-ever strokes in a defined population of inner London, including 22 electoral wards in Lambeth and Southwark. The total source population of the SLSR area was 357 308 individuals in the 2011 Census; their self-reported race was as follows: 56% white, 25% black (7% black Caribbean, 14% black African, and 4% black other), and 18% of other ethnic backgrounds. All individuals are included in the analyses of temporal trends in recurrence and mortality. Individuals recruited to the cohort between January 2000 and December 2018 were included in the analyses of stroke subtype, a subgroup of patients from 2005 onwards were included for analyses of poststroke care. Individuals were followed up at 3 months and then on an annual basis.

### Case Ascertainment

Standardized criteria were applied to ensure completeness of case ascertainment, including multiple overlapping sources of notification. All patients with a suspected diagnosis of stroke or transient ischemic attack documented in different hospital and community-based information sources were investigated for study eligibility. Patients admitted to hospitals serving the study area were identified by regular reviews of acute wards admitting patients with stroke, weekly checks of brain imaging referrals, and monthly reviews of bereavement officer and bed manager records. Additionally, national data on patients admitted to any hospital in England and Wales with a diagnosis of stroke were also screened for additional patients. To identify patients not admitted to hospital, all general practitioners within and on the borders of the study area were contacted regularly and asked to notify the SLSR of patients with stroke. Regular communication with general practitioners was achieved by telephone contact and quarterly newsletters. Referral of nonhospitalized patients with stroke to a neurovascular outpatient clinic (from 2003) or domiciliary visit to patients by the study team was also available to general practitioners. Community therapists were contacted every 3 months. Death certificates were checked regularly. Completeness of case ascertainment has been estimated at 88% by a multinomial-logit capture-recapture model using the methods described elsewhere.^17^

### Definitions

Stroke was defined according to the World Health Organization criteria; *International Classification of Diseases*-*Tenth Revision* codes I60.-, I61.-, I63.-, and I64 were included. As per this definition, patients who died within 24 hours of stroke were included but those who had an event lasting less than 24 hours were classified as TIAs and, therefore, excluded from the register. Stroke recurrence was defined by the same criteria as the initial stroke. Both ischemic and hemorrhagic stroke recurrences were recorded. Only recurrences 21 days after the initial event, or if earlier, clearly in a different vascular territory were included.^18^

Pathological stroke subtype was defined by brain imaging, lumbar puncture, or postmortem autopsy. The proportion of patients who received any brain imaging ranged from 95% in 2000 to 2003 to 100% to date. Neuroradiology or necropsy results classified strokes into ischemic stroke, primary intracerebral hemorrhage, or subarachnoid hemorrhage. Ischemic strokes were further investigated according to 2 stroke subtypes: The Oxfordshire Community Stroke Project classification^19^ and the modified TOAST classification (Trial of ORG 10172 in Acute Stroke Treatment).^20^ The Oxfordshire Community Stroke Project contains 4 subtypes according to clinical features: lacunar infarct, total anterior circulation infarcts, partial anterior circulation infarcts, and posterior circulation infarcts. Modified TOAST denotes 5 pathogenetic subtypes: large artery atherosclerosis, cardioembolism, small-vessel occlusion (SVO), other determined pathogenesis and no pathogenesis identified. Data with modified TOAST classification were collected only after the year 1999. Additionally, we classify hemorrhagic strokes as primary intracerebral hemorrhage or subarachnoid hemorrhage. Assignment to each classification was done by the senior stroke consultant acting as clinical lead on the study. Classification was based on clinical history, brain imaging (computed tomography and magnetic resonance imaging), vascular imaging (carotid doppler ultrasound, computed tomography angiography, magnetic resonance angiography), cardiac monitoring, and echocardiography. In our study cohort, 95% of patients had a brain scan (90% computed tomography, 25% MRI), 63% had at least one modality of vascular imaging, 81% had cardiac monitoring, and 40% had echocardiography.

The SLSR received notification of deaths and associated death records from National Health Service digital providing the date and cause.

### Data Collection

At the time of the index stroke, information was collected by highly trained fieldworkers contemporaneously from medical records and complemented with patient interview. Information was collected on diagnoses before the stroke of hypertension, TIA, atrial fibrillation, MI, diabetes mellitus, and other medical conditions not considered in this analysis, treatments received in hospital and prescriptions at discharge. Attendance by a specialist stroke physician during hospitalization and whether they were seen in a stroke unit was recorded.

### Ethics

Written informed consent and assent, when appropriate, were obtained from all participants or from a relative for the participants who were too impaired to provide written consent. Ethical approval for the study was obtained from the ethics committees of Guy’s and St Thomas’ Hospital Trust, King’s College Hospital, Queens Square, and Westminster Hospital (London).

### Statistical Methods

A description of the cohort at the time of their first stroke and a comparison of stroke subtype between initial stroke and recurrent stroke is reported using frequencies and percentages. The cumulative risk of recurrence and the combined outcome of recurrence or death occurring by 3 months, 1-, 5-, and 10-years were produced from the failure function using Kaplan-Meier estimates. Cumulative risk is presented overall, by year group and stroke subtype. The cumulative incidence of recurrence is also reported using a competing risks analysis to account for mortality before the event. Associations between risk factors and outcome were tested across the full follow-up period using cox proportional hazards models adjusting for all baseline factors considered. The risk factors considered were prespecified, and all were included in adjusted regression models. To assess the effect of subtype, we include only index strokes since 2000 adjusting for all covariates. Index strokes occurring since 2005 are used to look at poststroke treatment where we do not adjust for demographics or prestroke risk factors due to small number of events. We repeat the analysis for recurrence-free survival analyzing the time to recurrence or death. Statistical analyses and graphics were performed using Stata Version 14.2.

## Results

A total of 6052 individuals were registered on the SLSR between January 1995 and December 2018. A third (n=2082, 34%) were under 65 years at the time of their first stroke and 2534 (42%) were over 75 years, 2940 (49%) are female, 1906 (31%) are of nonwhite ethnicity. Seventy-one percent of first strokes (4326) were ischemic, and the most prevalent risk factor before the stroke was hypertension (3760, 65%). Further details of the population are presented in Table 1.

**Table 1.**
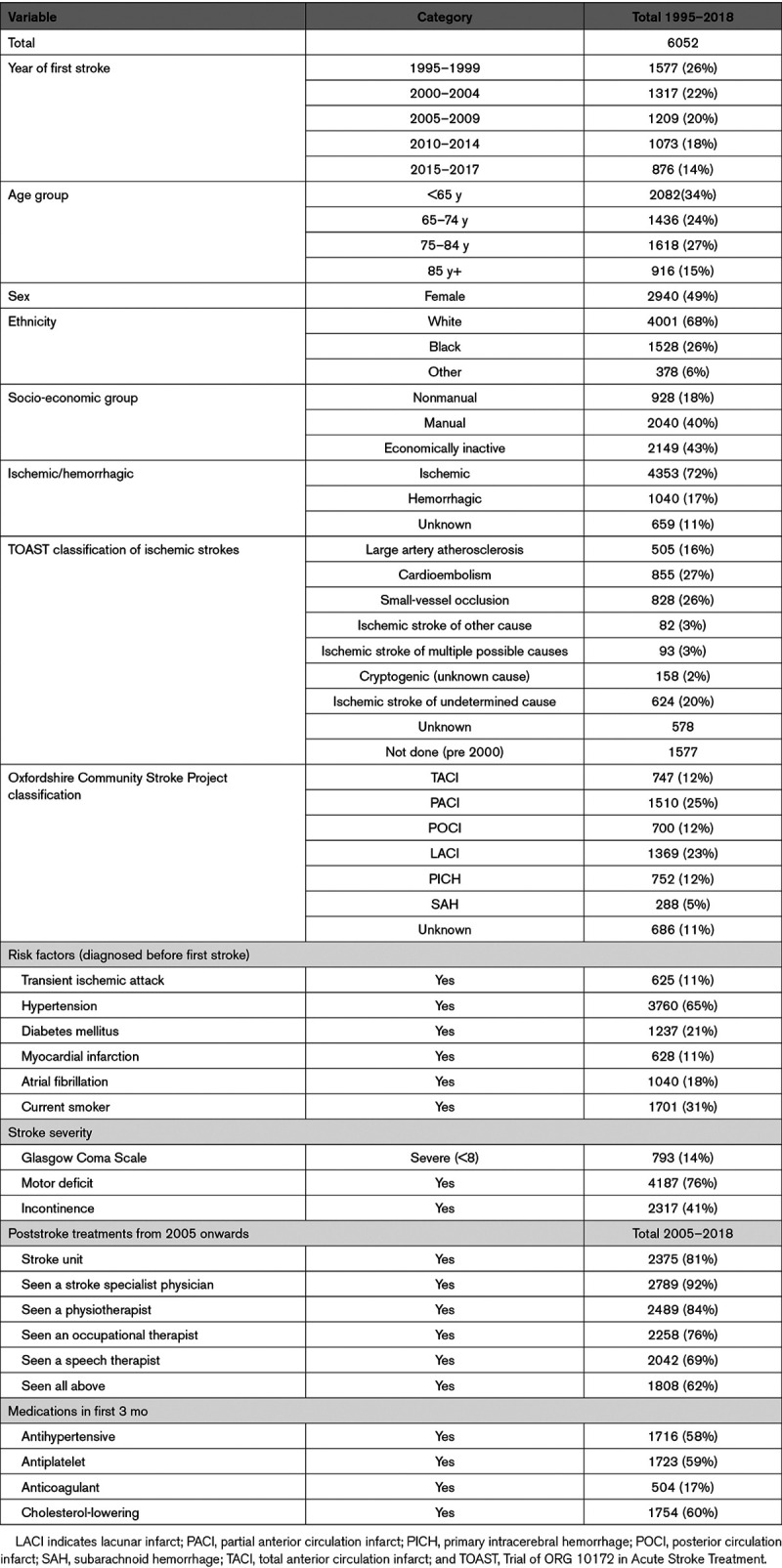
Description of Sample at Baseline (Stroke Onset)

### Stroke Recurrence

During follow-up 650 individuals had at least one stroke recurrence (21.4 per 1000 person-years), including both ischemic and hemorrhagic. The median follow-up time for the stroke recurrence analysis was 3.0 years (range: 1 day to 22·5 years) with a total of 30 412 person-years of follow-up. The cumulative risk of a stroke recurrence decreased between 1995 and 2005 with little change since (Table 2, Figure [A]). Risk of recurrence at 5 years was 18% (95% CI, 15%–21%) for first strokes between 1995 to 1999 dropping to 12% (10%–15%) for first strokes between 2000 and 2004 and remaining at around 10% (9%–13%) for first strokes since 2005. When accounting for death as a competing risk the 5-year recurrence rate is lower at 8.7% (8.0%–9.4%) and the 10-year recurrence at 11.2% (10.4%–12.1%).

**Table 2.**
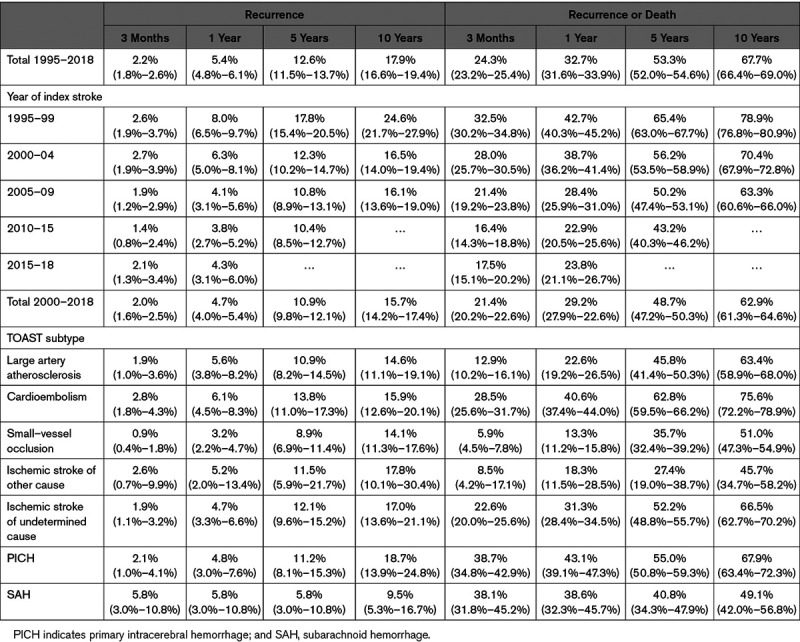
Cumulative Incidence of Recurrence and Recurrence or Death

**Figure.**
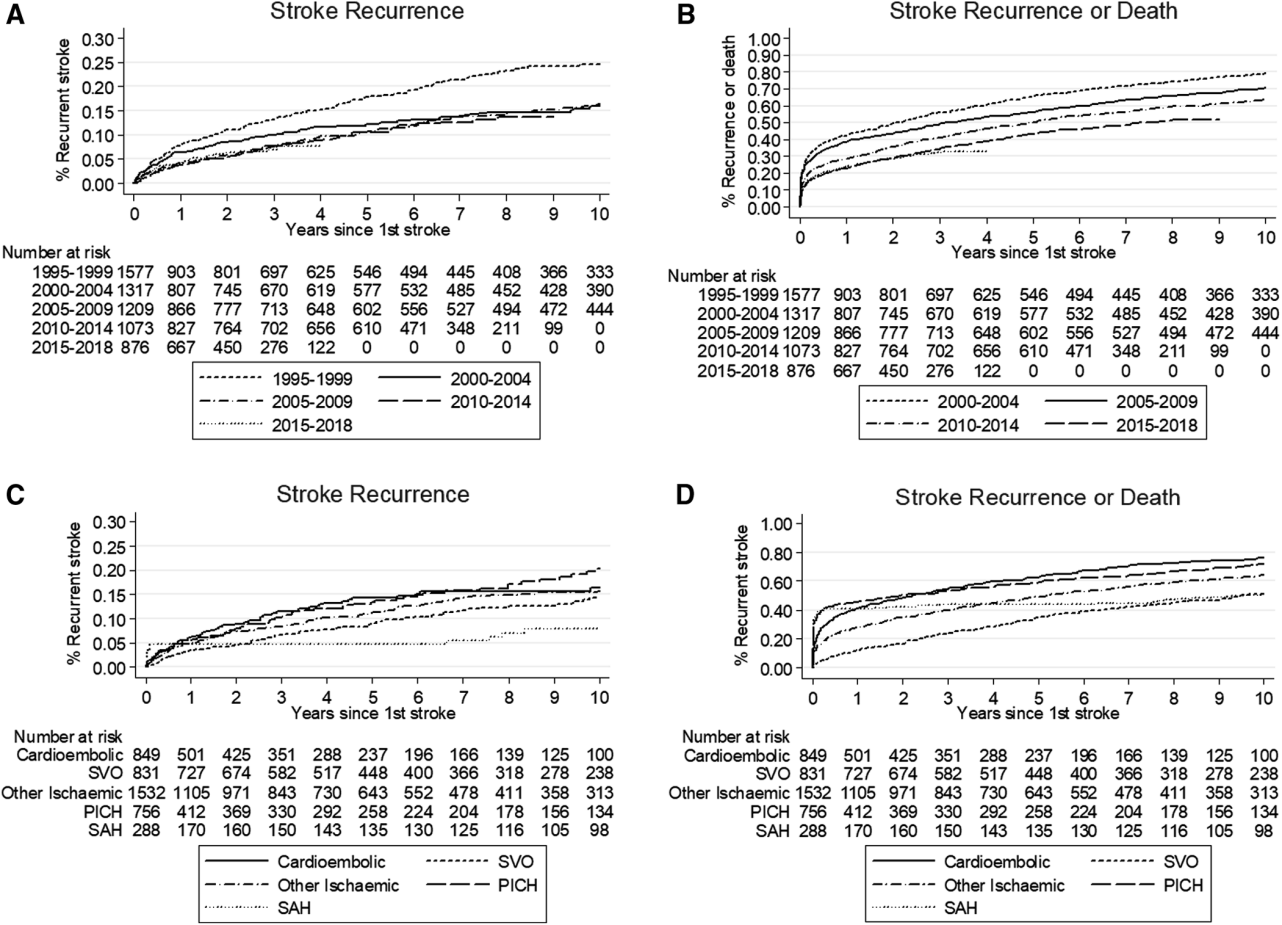
**Failure function for recurrence and recurrence-free survival by year and subtype.** **A**, Stroke recurrence by year of first stroke; (**B**) recurrence-free survival by year of first stroke; (**C**) stroke recurrence by type of stroke; and (**D**) recurrence-free survival by type of stroke.

### Recurrence-Free Survival

During the follow-up period, 4035 individuals had a recurrence or died (133 per 1000 person-years). The median follow-up time for the stroke recurrence analysis was 3·0 years with a total of 30 402 person-years of follow-up. The cumulative risk of recurrence or death by 5 years has reduced from 65% (63%–68%) in 1995 to 1999 to 43% (40%–46%) for first strokes occurring in 2010 to 2015.

### Stroke Subtypes as a Risk Factor

Etiologic TOAST stroke subtypes were collected since 2000 and so further analysis by type is restricted to this time period. There is no evidence of an association between TOAST subtype and recurrence in either crude analysis (Table 2), adjusted for prestroke risk factors and stroke severity (Table 3) or accounting for death as a competing risk (data not shown). When considering the combined outcome of recurrence or death individuals with a cardioembolic stroke have the worst prognosis (63%, 59%–66% recurrence or death by 5 years, Table 2 unadjusted). Risk of recurrence or death is higher for cardioembolic (hazard ratio [HR], 1.48 [1·25–1.74]), large artery atherosclerosis (HR, 1.34 [1.13–1.58), and primary intracerebral hemorrhage (HR, 1.29 [1.06–1.55]) as well as for undetermined ischemic strokes (HR, 1.42 [1.21–1.66]) compared with SVO after adjustment for prestroke risk factors and severity (Table 3).

**Table 3.**
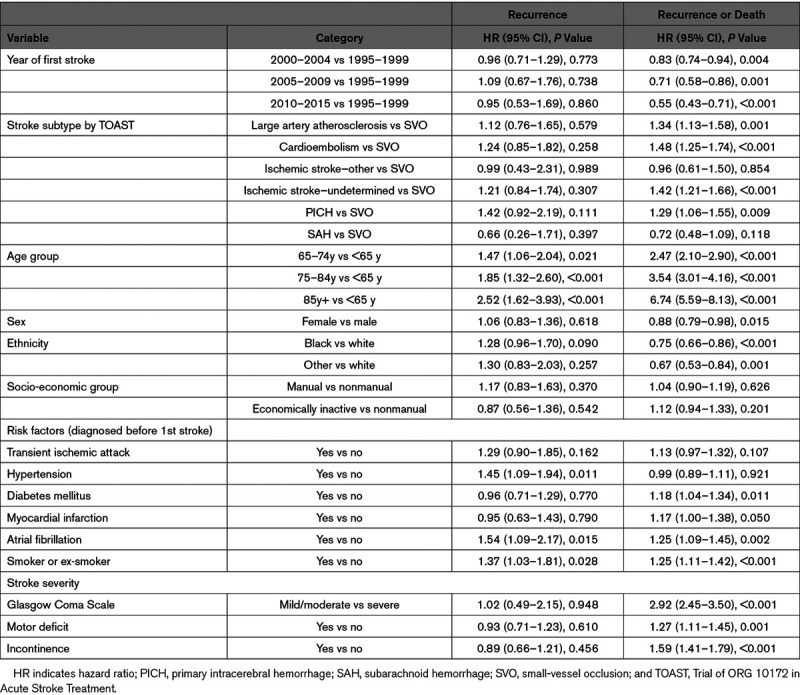
Prestroke Risk Factors for Stroke Recurrence Including TOAST Classification

Risk of recurrence seems to be reducing in large artery atherosclerosis and ischemic stroke of unknown cause since 2000 while remaining at a similar level over time in cardioembolic and SVO strokes. However, there is no significant interaction between subtype and time indicating that there is no evidence of a difference in time trends between subtypes.

Overall, comparing ischemic to primary intracerebral hemorrhage and subarachnoid hemorrhage strokes indicates that there is no difference between them in terms of recurrence rates but subarachnoid hemorrhage strokes have the best recurrence-free survival with no difference observed between ischemic and primary intracerebral hemorrhage (Figure).

The largest proportion of first recurrent strokes are the same subtype as their index stroke (Table 4). However, only for cardioembolic stroke and hemorrhage are the majority of first recurrences of the same type (54% and 51%, respectively). There is variation between the index and recurrent stroke subtype, only 26% of large artery atherosclerotic index strokes that have a recurrence are of the same classification and 35% of SVO index strokes have an SVO recurrence (Table 4).

**Table 4.**
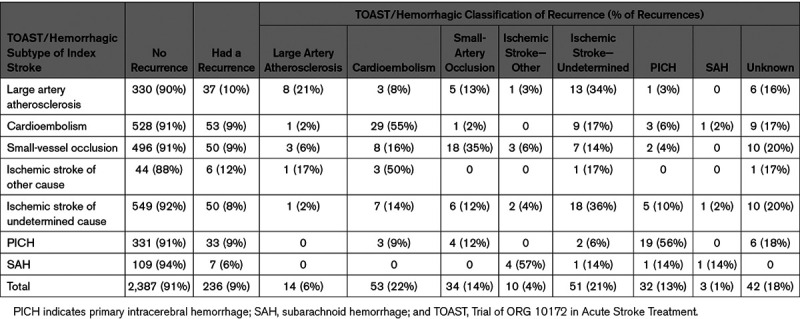
Recurrence TOAST/Hemorrhagic Classification by Index TOAST/Hemorrhagic Classification, 2000–2018

### Other Stroke Risk Factors

Table 3 shows that older age, prior atrial fibrillation, prior hypertension, and smoking are risk factors for a second stroke after full adjustment. There is a suggestion that being of black ethnicity increases the risk of recurrence compared with white patients but is associated with a reduced risk of recurrence or death compared with white ethnicity (HR, 0.74 [0.65–0.84]). We observe no evidence that having had a myocardial infarction before the index stroke or the severity of stroke are associated with risk of recurrence but they are associated with risk of the combined recurrence or death outcome.

In subgroup analyses of index strokes occurring since 2005, although being treated in a stroke unit, seen by a stroke specialist and secondary prevention medications tend to suggest a reduced risk of recurrence only antithrombotics show a statistically significantly association (Table 5). There is little change in the type of strokes seen over time, though there is a suggestion of an increase in the proportion of cardioembolic strokes in the past 5 years (Figure I in the Data Supplement). Prescription of secondary preventions medications as well as treatment by a stroke specialist and in a stroke unit are associated with a reduced risk of the joint outcome recurrence or death. These results are minimally adjusted for age, Glasgow Coma Scale, and prestroke risk factors as the number of events limits the variables that can be included. There has been little change in secondary prevention prescription rates since 2005 (Figure I in the Data Supplement). Prescription of antithrombotics has stayed at around 70%, antihypertensives and statins at around 60%.

**Table 5.**
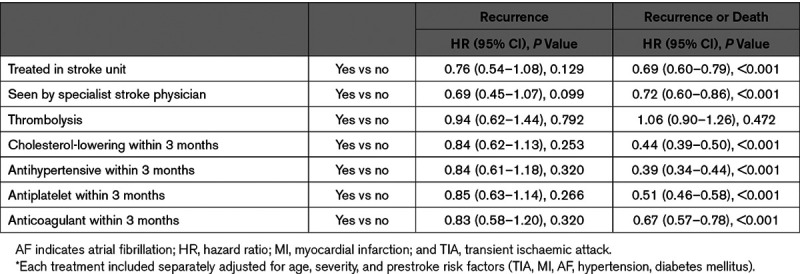
Poststroke Treatment Associated With Recurrence and Death, 2005 Onwards

We also find that the increased risk of recurrence or death due to having a previous diagnosis of atrial fibrillation remains after adjustment for treatments received. Of the 488 individuals who had been diagnosed with atrial fibrillation before their index stroke and reached discharge from hospital, 123 (25%) were prescribed anticoagulants and no antiplatelets at discharge or within 3 months, 154 (32%) were prescribed antiplatelets and no anticoagulants at discharge or within 3 months and 52 (11%) received both. One hundred fifty-nine individuals (33%) received neither.

## Discussion

We have extended previous SLSR reports^18, 21^ by estimating trends over the past 20 years in stroke recurrence and explored associations with different exposures including demographics and stroke etiologic subtypes. Recurrence rates after first strokes reduced between 1995 and 2005 but have remained at approximately the same level since then. Recurrence-free survival has continued to improve, driven mainly by a reduction in mortality. Although cardioembolic or hemorrhagic subsequent strokes are largely preventable, the majority of recurrences occurring after these strokes are the same subtype.

The reduction in recurrence seen at the beginning of our study suggests that secondary prevention measures have been effective, and recurrences are now being maintained at a low level, a trend reported in other studies which also observed only small reductions since 2000.^22^ Despite this flattening in recurrence rates the joint outcome of recurrence-free survival continues to improve. Since this is not driven by stroke recurrences, it may be that general health is improving or that treatment of prevalent vascular comorbidities such as MI are continuing to improve.^23^

Alternatively, those with a cardioembolic or hemorrhagic stroke who have a recurrence are most likely to have a repeated stroke of the same type, in our study sample. This same trend was reported in the SLSR previously, but now with a larger sample size.^21^ Further exploration of the subgroup that have difference recurrence subtype to their index stroke is not possible due to limited numbers. This may suggest that the secondary prevention measures are not having the impact we would expect, maybe due to low coverage, complication with these vascular subtypes or problems with adherence. A previous SLSR analysis found 60% of patients with stroke with AF were not treated with anticoagulants^11^ and more recent reports suggest that this figure is 46% across the United Kingdom.^12^ Here we have observed that 33% of patients with stroke with a previous diagnosis of AF do not receive either anticoagulants or antiplatelets at discharge. This is a particular concern as we find that having AF is one of the main indicators of a poor outcome after stroke, a finding reiterated in other studies.^24,25^ Patients with stroke are also failing to receive treatment for other health issues, for example only 60% to 70% of those with hypertension receive the appropriate treatment and 70% to 75% of those with diabetes mellitus.^11^ Ensuring treatment for these patient groups could have a positive impact on their outcomes, though this has not been tested explicitly in this study. In addition, there may be issues with adherence, we only have information on prescription and so cannot analyze this further; but it is likely that we will underestimate the true effect of taking the medication. It is hypothesized that patient adherence and the success of secondary prevention strategies may be improved by early initiation in hospital.^26^ This is supported indirectly by our finding that those who are seen by a specialist stroke physician or in a stroke unit have better survival though we do not see a significant association with recurrence.

Having a previous diagnosis of AF is a risk factor for a second stroke, supporting findings of a Swedish cohort.^25^ There is some evidence that being of black ethnicity increases your risk of recurrence compared with patients with stroke of white ethnicity, though the opposite is true when it comes to the joint outcome of recurrence or death. In the US black minority groups, having a stroke are less likely to receive expected acute interventions compared with their white counterparts as well as having increased mortality.^27^ However, analyses of the SLSR data found the opposite, that Caribbean/African stroke patients were more likely to be admitted to hospital or to a stroke unit.^11^

There are many strengths in our study. It is a large study of patients with stroke with long-term follow-up, both the primary outcome recurrence is captured through the stroke register as well as by follow-up of patients and is well completed. The risk factor analysis was conducted in a large cohort, though only those with complete information on all covariates could be included.

We have considered only baseline risk factors of recurrence, further analyses investigating the impact of secondary prevention measures after discharge and adherence would be of interest. This would benefit from linking to other resources such as primary care and cardiovascular disease registers to get a more accurate presentation of other cardiovascular outcomes such as myocardial infarction.

In conclusion, the rate of recurrence reduced until 2005 but has not changed since. The majority of cardioembolic or hemorrhagic strokes recurrences are of the same subtype suggesting that the implementation of effective preventive strategies as detailed in the National Clinical Guidelines for Stroke is suboptimal in these stroke subtypes.

## Acknowledgments

We thank patients, their families, and the fieldworkers who have collected data for the South London Stroke Register since 1995.

## Sources of Funding

This work was supported by funding from the National Institute for Health Research (NIHR) Applied Research Collaboration (ARC) South London at King’s College Hospital National Health Service Foundation Trust and the Royal College of Physicians, as well as the support from the NIHR Biomedical Research Centre based at Guy’s and St Thomas’ National Health Service Foundation Trust and King’s College London.

## Disclosures

None.

## Supplementary Material


