# Supplementary material for: Risk and Secondary Prevention of Stroke Recurrence: A Population-Base Cohort Study
Source: Stroke. 2020 Jul 10;51(8):2435–44. doi: 10.1161/STROKEAHA.120.028992 (PMC7382537; doi:10.1161/STROKEAHA.120.028992)

SUPPLEMENTAL MATERIAL

Supplemental Figures

Supplementary Figure I: trends over time in subtypes and medications

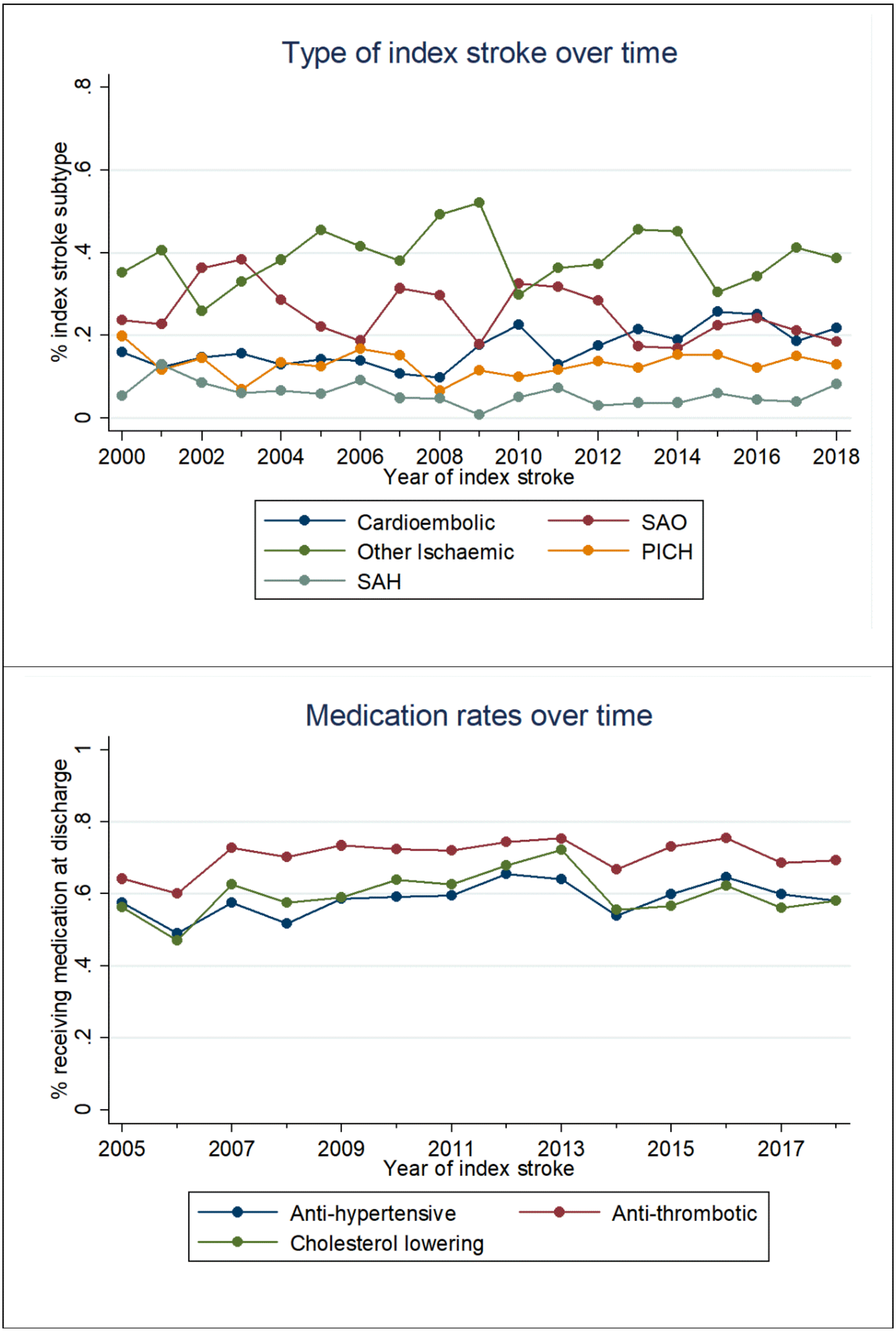

Supplement: Supplementary file 1 [file str-51-2435-s001.pdf]
